# The nordic maintenance care program: patient experience of maintenance care—a qualitative study

**DOI:** 10.1186/s12998-021-00388-z

**Published:** 2021-08-02

**Authors:** Jesper Hjertstrand, Per J. Palmgren, Iben Axén, Andreas Eklund

**Affiliations:** 1 Unit of Intervention and Implementation Research for Worker Health, Institute of Environmental Medicine (IMM), Karolinska Institutet, Nobels väg 13, Solna, 171 77 Stockholm, Sweden; 2 Department of Learning, Informatics, Management and Ethics, Karolinska Institutet, Widerströmska huset, Tomtebodavägen 18A, Solna, 171 65 Stockholm, Sweden; 3 ELIB, The Norwegian Chiropractic Research Foundation, Et liv i bevegelse, Oslo, Norway

**Keywords:** Chiropractic, Maintenance care, Low back pain, Qualitative study, Purposeful and maximum variation sampling strategy, Semi-structured interviews, Inductive approach

## Abstract

**Background:**

Low back pain is one of the major causes of disability world-wide. Most back pain sufferers experience pain that is recurrent or persistent, making management of this condition a priority. In a series of previous studies, chiropractic maintenance care has been found to be an effective way of reducing the number of days with pain, particularly for patients with a certain psychological profile. However, little is known about patients’ experience of this kind of management plan. This study aimed to explore patient experiences and preferences by looking at barriers to and facilitators of engaging in and maintaining a care plan, and to contrast the data using psychological sub-groups.

**Methods:**

In this qualitative study we performed semi-structured interviews with 24 patients who had previously participated in a Swedish trial evaluating maintenance care. They were purposefully selected to obtain richness, variation and breadth of data. The data were analyzed using inductive qualitative manifest and latent content analysis. We used the theory of planned behavior to deepen our understanding of the constructed themes.

**Results:**

The analysis resulted in two overarching dimensions: “when maintenance care is of high value” and “when maintenance care is of low value”. Four factors were jointly identified as obstacles to maintenance care by patients in all the psychological subgroups. These factors were: *Cost demanding, A sense of low value, Perceived as unavailable* and *Fear of treatment.* The one factor seen as facilitating maintenance care by patients in all the subgroups was *Care that is patient-centered.*

**Conclusions:**

The findings reveal a variance of both positive and negative experiences of MC in the psychological subgroups. These findings can deepen our understanding of how patients experience MC and can help clinicians to understand when patients might regard maintenance care as being of high value.

**Supplementary Information:**

The online version contains supplementary material available at 10.1186/s12998-021-00388-z.

## Background

Low back pain (LBP) is a common, highly recurrent and disabling condition with very great socioeconomic implications. It therefore seems logical to focus available research resources on prevention [1, 2]. Despite previous trials having investigated a wide variety of treatments for LBP, the evidence for prevention is scarce [2]. Currently, the most promising intervention for secondary prevention of LBP is exercise and education [3]. However, for some patients a more structured framework of on-going support and pain management may be required [2].

There is emerging evidence supporting the use of pre-planned supportive long-term manual care, so called Maintenance Care (MC) for a certain group of patients with recurrent and persistent LBP [4–7]. Chiropractors have described the procedure as “a type of prolonged care delivered at regular intervals” or as “a preventive approach, aimed at preventing new episodes and maintaining improvement” [5]. In a strategic research program, the Nordic Maintenance care program has been investigated in a series of projects with the aim of understanding the procedure and establishing effectiveness and cost-effectiveness [4–18]. Patients defined as “Dysfunctional” by the West-Haven Multidimensional Pain Inventory [19, 20], with high pain severity, marked interference with everyday life, high affective distress, low perception of life control and low activity levels, report clinically significant improvements from MC with no or little additional cost [4, 6, 7]. These patients report fewer days with activity limiting pain, less acute pain episodes and longer pain free periods if treated with MC compared to patients treated on a need-basis [4, 6, 7].

Studies among Scandinavian chiropractors reveal that around 22–41% of all visits are dedicated to MC. Treating patients regularly may also reflect the persistence and recurrent nature of many musculoskeletal conditions. The majority (98%) of Swedish clinicians believe that MC is a useful procedure for some patients [5].

Bringsli et al. investigated patients’ perceptions of their MC care plan in an anonymous survey and found that the purpose was to prevent pain, to remain as pain free as possible, and to prevent disease in general. In addition, most of the patients felt those goals were achieved to a high degree [11]. Quantitative introspections can provide useful information about patients’ perceptions, but they offer only limited insight into the intricacy of their experiences. Besides the study by Bringsli and colleagues [11], little is known about patients’ experiences and preferences or about factors which facilitate or hinder MC. To our knowledge there are no existing qualitative explorations of how patients in these contexts experience MC and what their preferences regarding this approach are. In other words, there is a paucity of scientific research examining this dimension of MC. Given the emerging evidence of the effectiveness of this procedure there is a clear need to understand the patient perspective and experience of the MC encounter. This information is important to facilitate high compliance, patient-centered care and the optimum effectiveness of the procedure before implementing the strategy on a large scale.

When introspecting the experiences and preferences of stakeholders such as patients about engaging in and maintaining a MC care plan, theories can be used to help us better understand contextual findings. Furthermore, we believe, in line with Morden et al. [21], that theoretical frameworks can help to broaden the perspective and better understand the phenomenon being studied. Theories can provide access to more complex phenomena by establishing the authenticity and plausibility of ideas and offering conceptual frameworks on which to build new knowledge. In order to understand the behaviors related to MC, the theory of planned behavior (TPB) has been used as a theoretical framework by which patients’ behavioral intentions can be observed and understood in a wider context [22]. Ajzen (1991) proposed a correlation between planned behavior and actual behavior [22] The correlation has been tested empirically and shown to be moderate (0.44–0.48) across different populations and behaviors [23, 24]. TPB assumes that a person’s behavior is deliberate and planned and can be predicted on the basis of three indicators/determinants of behavioral intention. First, favorable attitudes toward the behavior and perception of likely consequences of the behavior (pros and cons regarding the outcome of the behavior); second, perceptions of the subjective/social norms which support the behavior (social pressure regarding the behavior perceived by the individual); and third, perceptions of behavioral controls that support the behavior (individual perception of the ability to perform the behavior). In this context TPB may be used as a theoretical framework to understand and aid implementation of the findings from this project in a clinical setting, with the aim of improving compliance and adherence to treatment-plan, recommendations and advice.

## Methods

### Aim

The aim of the study was two-fold: (1) to explore patients’ experiences and preferences regarding MC, with an emphasis on barriers to and facilitators of engaging in and maintaining a care plan; and (2) to contrast the interview data using three psychological subgroups: adaptive copers, interpersonally distressed, and dysfunctional, as defined by the Swedish version of the West Haven-Yale multidimensional pain inventory.

### Study design and methodology

The present study was part of a larger project employing a prospective mixed-methods approach anchored in a pragmatic research tradition, as outlined by Creswell [25]. It was conducted within an interpretative paradigm, according to which knowledge is viewed as relative and socially constructed [25]. In line with this view, there was an underlying assumption that, rather than endeavoring to reveal an objective and ‘‘real’’ truth, findings result from an interplay between the phenomenon under scrutiny and the investigators [26]. We considered a qualitative approach appropriate to explore human experiences [27]. The study was informed by the TPB, by framing the phenomenon under study but, above all, as a lens to further comprehend emerging findings. However, the TPB was not used specifically to formulate the research questions or to create a priori coding for the analysis but rather to be used as a lens to understand and conceptualize the phenomenon under study.

### Context and participants

In Sweden, chiropractors are licensed healthcare practitioners under the National Board of Health and Welfare (Socialstyrelsen). For the past 20 years, clinical research has been conducted by chiropractors in a nationwide practice-based research network (PBRN) [28]. Between 2012 and 2016, 35 members of the Swedish Chiropractic Association (part of this PBRN), collected data on patients with recurrent and persistent LBP for the randomized clinical trial investigating the effectiveness and cost-effectiveness of MC [4, 6–8] which was described in the introduction. Patients who had been randomized to the intervention MC were contacted in January and February 2020 based on a planned sampling scheme.

A purposeful and maximum variation sampling strategy was employed to obtain richness and variation in the data [29]. A wide range of presumptive responders was sought to give a broad diversity of gender, age and psychological profile. Therefore, participants were identified and selected by the principal investigator (AE) in several consecutive steps based on earlier empirical data [4, 6–8]. Sixty-three potential participants were invited to participate in the study, 39 of whom declined on personal grounds or failed to reply to the initial contact, leaving a total of 24 participants eligible for the study. Our sample comprised these 24 individuals, 12 females and 12 males. Their characteristics are presented in Table 1. The participants were contacted via text message and later phoned to schedule virtual face-to-face interviews using the Zoom platform. Information about the study was sent by email. Prior to the interview, the participants were able to ask questions about the study. Informed consent was obtained verbally. Written informed consent was also obtained, and full confidentiality was guaranteed. Participation was voluntary, and the respondents were informed that they could withdraw at any time without giving a reason.

**Table 1:** Descriptive data of participants in study

Participant	Age	Sex	MPI	RMDQ	TDP	Professional background
1	32	M	ID		54	Office worker, IT
2	41	M	ID	1	146	Sheet metal/construction worker
3	42	M	ID	7	207	Office worker, sales
4	45	M	ID	2	66	Construction worker
5	37	F	ID	3	132	Office worker, IT
6	41	F	ID	9	49	High school teacher
7	44	F	ID	6	71	Office worker, IT
8	50	F	ID	5	51	Bank clerk
9	31	M	DYS	10	115	Carpenter
10	38	M	DYS	4	193	Painter
11	44	M	DYS	11	67	Service manager, warehouse
12	48	M	DYS	10	24	Workshop manager/lorry driver
13	24	F	DYS	4	28	Student, nurse
14	32	F	DYS	4	26	Office worker, biologist
15	52	F	DYS	2	6	Care assistant
16	53	F	DYS	5	103	Office worker, accounting
17	33	M	AC	4	22	Warehouse worker
18	38	M	AC	1	36	Shop assistant
19	52	M	AC	6	125	Operations technician
20	62	M	AC	5	25	Store manager
21	34	F	AC	0	28	Actor
22	36	F	AC	0	65	Administrator, social services
23	43	F	AC	1	37	Bank clerk
24	51	F	AC	6	50	Nurse

Age, Participant age at RCT baseline; M, Male; F, Female; MPI, West Haven-Yale Multidimensional Pain Inventory subgroup classification at initial screening visit during RCT; ID, Interpersonally Distressed profile; DYS, Dysfunctional profile; AC, Adaptive Coper profile; RMDQ, Roland Morris Disability Questionnaire (0–24, at RCT baseline); TDP, Total number of days with activity limiting low back pain during RCT study period (52 weeks)

### Data collection

The data were collected by means of individual semi-structured interviews to capture patients’ experiences, opinions, feelings, and knowledge [29]. An interview guide was constructed (Additional file 1: Appendix 1) on the basis of two criteria: the interview should (1) correspond to the aim of the study, and (2) using empirical findings from the scientific literature [4, 6, 7, 30, 31], it should help to identify potential challenges patients face when considering MC as a preventive strategy for their condition. It should also describe the potential facilitating factors or perceived benefits of the care plan. The interviewer (JH) had previous experience of conducting qualitative interviews. The interviews were conducted virtually, each lasting on average 33 min (minimum–maximum 18–60 min). The audiotaped interviews yielded a total of 789 min of recorded material, which was transcribed verbatim by someone independent of the study. This resulted in a total of 345 pages (on average 14 pages per participant) of textual data. All interviews were conducted, transcribed, and analyzed in Swedish. Once the analysis was completed the main findings consisting of qualitatively labeled textual content with accompanying supportive quotes, were translated into English. During multiple consensus meetings the translated material was discussed, and the condensed English translations were compared to the Swedish verbatim translations to ensure the appropriate meaning was captured.

### The West Haven-Yale multidimensional pain inventory (MPI)

At the initial screening visit for the RCT the participants recruited for the trial were assessed using the Swedish version of the West Haven-Yale Multidimensional Pain Inventory (MPI-S). MPI was developed to assess the cognitive-behavioural aspects of the pain experience and can be used to classify patients into psychological subgroups. The MPI instrument has been shown to have acceptable reliability and validity [32–34] and has been used in a variety of populations suffering from conditions such as neck pain and LBP [35–37], temporomandibular disorders [38], headaches [39], fibromyalgia [40] and cancer pain [41]. The instrument has been validated across cultures and translated into several different languages [42–44].

The MPI is divided into two parts consisting of 34-items and 8-scales in total. Five *psychological* constructs (pain severity, interference, life control, affective distress, and support) and three *behavioral* constructs associated with individuals in close relationships with the patient (punishing responses, solicitous responses, and distracting responses) are measured. Based on the scores of the instrument, three psychological subgroups have been identified [19, 32, 45] and have been named adaptive copers, interpersonally distressed, and dysfunctional [20]. These subgroups have been replicated in several studies and have been found to predict treatment outcome [4, 6, 34, 35, 46] and sick leave [36, 47] and are thought to have clinically meaningful properties.

Patients classified as adaptive copers are characterized by low pain severity, low interference with everyday life, low life distress, a high activity level and a high perception of life control. Out of the three subgroups these individuals have the best prognosis and the lowest risk of long-term sick-leave [36, 47–49]. Individuals classified as interpersonally distressed report challenges in close relationships and often describe distrust of others whom they view as responsible for their problems. Often, their spouses or significant others respond negatively to their pain behaviour by not being supportive/helpful or expressing irritation, frustration, and anger. Compared to the adaptive coper subgroup, the interpersonally distressed individuals have a poorer prognosis and a higher risk of long-term sickness absence [47]. The dysfunctional sub-group, on the other hand, reports high pain severity, which interferes with everyday life, and high affective distress, low perception of life control and low activity levels. Pain-avoidant coping strategies (e.g. catastrophizing) and fear and avoidance of activities related to pain are commonly reported among these individuals [20]. Compared to the other two subgroups, the dysfunctional individuals have the worst prognosis and the highest risk of long-term sickness absence [47].

### Data analysis

The data were analyzed using an inductive approach to conventional qualitative content analysis [30, 50, 51], primarily informed by the method outlined by Graneheim and Lundman [30]. The transcripts were examined line-by-line, and subcategories and categories were developed without predetermined coding schemes. The analysis comprised several steps: (1) the transcribed interviews were read initially by JH to become familiar with the text; (2) the textual data were read and analyzed by all authors, both separately and together; (3) the investigators jointly identified meaning units relating to the aim of the study and the questions in the interview guide; (4) the meaning units were discussed and condensed, and codes for the phenomenon under investigation were created by JH, PJP and AE; (5) interpretative cross-contrasting of subcategories and categories were performed; and (6) a primarily interpretational analysis was carried out, i.e., the investigators went beyond the explicit manifest content. Subcategories and categories were interpreted and explored into themes and overarching dimensions expressing the underlying latent content of the data [30].Thus, the analysis focused on interpreting the meanings in the text, with the transcripts subjected to both manifest and latent content analysis. One way to understand these concepts is to relate them to one of the tentative axioms in communication theory, as described by Watzlawick et al. [36] This suggests a depiction of the manifest content as what the text explicitly says, dealing with the surface structure and the most obvious meanings of the text. Conversely, the latent content is subjected to an interpretative reading of what the text implicitly talks about and captures the deep structural meanings conveyed. In a second step, the three data sets deriving from the different participant profiles (adaptive copers, interpersonally distressed, and dysfunctional) were cross-contrasted with regards to emerged categories, themes and dimensions. The original interviews were conducted without considering the psychological classification and all interviews were based on the same interview guide. The psychological subgroup classification was used as a raster to view the data through after the main analysis to see if the different subgroups had fundamentally different perspectives. The entire analytical process was discussed and adjusted until a consensus was reached among the investigators. Although the above steps might seem to be sequentially ordered, the analytical process and search for patterns was rather dynamic, iterative, and recursive. No software program was used to aid the analysis.

The issue of methodological rigor was addressed in various ways. The trustworthiness of the analysis was enhanced by investigator triangulation (investigators with different professional backgrounds; JH, undergraduate chiropractic student; PP, full-time researcher and educator; AE, part-time researcher and part-time clinician). Throughout the analytical process, and primarily due to the senior investigator’s prior understanding of the empirical context, constant comparisons between the subcategories and categories and the original data transcripts were made to ensure a good fit between the data and findings. We thus gave careful consideration to Patton’s dual criteria of internal homogeneity and external heterogeneity [29].

### Ethical considerations

Information about the study was sent by email to participants who had agreed to participate. They were then given further information about the study verbally. Participation was voluntary, and the informants were informed that they could withdraw at any time. Informed consent was obtained from the participants prior to the interviews, and full confidentiality was guaranteed. None of the information collected was identifiable, thus ensuring data anonymity. The study was conducted according to the tenets of the World Medical Association Declaration of Helsinki and approved by the Regional Ethical Review Board in Stockholm (Dnr 2019-04505).

## Results

### Facilitating factors

The analysis resulted in seven categories and three themes (Table 2) which describe the participants’ experience of factors which facilitate engaging in and maintaining a MC plan. Each theme is presented using the underlying categories and illustrated by supporting quotes. The overall dimension described circumstances that contribute to *When maintenance care is of high value*.

**Table 2:** Perceived facilitating factors for maintaining and engaging in a maintenance care plan

Subcategories	Categories	Themes	Dimension
It made my pain go away	Free of pain—moving and performing better	Care that improves quality of life!	When maintenance care is of high value
Enables me to stay well over time			
My physical abilities have improved			
Stimulated healthier behaviors			
Allows me to enjoy life	Makes me feel great!		
Helps me with my emotions, thoughts and boosts my self confidence			
Avoiding sick-leave	I don´t want to be off work		
Being more productive at work			
Readily available care	It fits into my life	Care that is structured, accessible and appreciated!	
Time efficient and effective treatment			
Small invested effort and no hassle			
Societal or employer reimbursement			
Regular visits offered continuity & motivation	A form of care: framework for regularity and support		
It created a feeling of reassurance			
Complements other health actions	Important piece of the puzzle		
A sense of professional, caring and personal relationship	The competent clinician providing for great doctor-patient rapport	Care that is patient-centered!	
Provided me with information, guidance and education			

#### Care that improves quality of life

This theme was interpreted by means of three categories: *Free of pain—moving & performing better, Makes me feel great!* and *I don’t want to be off work.* These are all components that, according to patients, have value when it comes to improved quality of life. Participants answered that pain relief offered them new possibilities, for example in terms of improved physical functioning, remaining well over longer time and that MC stimulated them towards healthier behaviors.

“Maintenance care can act as a springboard to start moving more, to start exercising, which, you see, can contribute to one’s overall health. This can impact the whole family. You might start eating and drinking healthier.”

(Participant 12, DYS).

The participants also said that MC improved their emotional state as well as self-efficacy and helped them enjoy life more. They also reported that MC allowed them to be more productive at work and avoid time off work.

”I would have never been able to continue my line of work if my back had felt the way it used to.”

(Participant 11, DYS).

“Well, apart from the physical aspect, not having pain, and, how shall I put it, being more confident that my back and body can handle the things I need to do, maintenance care has also helped my mental state. To know that I can carry, play and have fun with my children, be able to participate in physical activities without having to be left on the side-line wondering whether I can do it has been invigorating mentally.”

(Participant 18, AC).

#### Care that is structured, accessible and appreciated

This theme comprised three categories: *It fits into my life*, *A form of care: framework for regularity & support* and MC being an *Important piece of the puzzle*. These categories represent care that is perceived as structured, accessible and appreciated. Participants mentioned that a facilitating factor for engaging in and maintaining a MC plan was that it was readily available in terms of treatment times and that it was accessible logistically. Similarly, patients felt that the MC approach offered continuity and motivation over time. Regular visits, or check-ups were viewed as positive and encouraging. Additionally, participants reported that the MC approach provided reassurance, that it was comforting to know that care was only a phone call or booking away.

”When the pain came back I knew I had an appointment booked which meant I got help fast. I thought this was reassuring and it felt sort of comforting. There was a period where I felt worse and wasn’t as active with my training. During that time, it was very comforting to know that I had my appointment booked in advance.”

(Participant 24, AC).

”It was nice to meet the clinician regularly to get some tips and also a form of follow-up. I don’t think this should be underestimated, I think it has real value.”

(Participant 3, ID).

Finally, participants viewed MC as an important component of their health care routine. It was perceived as a complement to other treatment modalities or positive health actions such as exercising or massage.

“I currently receive a massage once a month which I also did during the maintenance care trial. In preventive terms, I felt like the massage complemented the chiropractic maintenance care very well.”

(Participant 21, AC).

#### Care that is patient-centered

Participants emphasised the importance of an *appropriate relationship with their clinician.* At the centre of this relationship was the ability of the chiropractor to be *professional and caring*.

”I would say that the clinician acted professionally and with care, which meant I felt trust and confidence in her ability as well as for the chiropractic profession in a way that I hadn’t before.”

(Participant 21, AC).

Additionally, patients highlighted related information and education as important facilitating factors for engaging in and maintaining a MC plan.

“The clinician showed me what was wrong with my back. I thought this was great at the start of treatment since I didn’t have that knowledge at the time. This education was very positive, he explained things in a clear way.”

(Participant 6, ID).

### Barriers

The analysis resulted in three themes regarding barriers to engaging in and maintaining a MC plan. Each theme was formulated as a question: *Does the benefit of maintenance care outweigh the cost?*, *Is maintenance care accessible?* and *Is maintenance care being delivered in a way that is congruent with a patient-centered perspective?* (Table 3). Each theme is described separately using the underlying categories as well as illustrative quotes. A further analysis, at the latent level, gave the final dimension *When maintenance care is of low value*, which encompasses the barriers to engaging in and maintaining a maintenance care plan.

**Table 3:** Perceived barriers to maintaining and engaging in a maintenance care plan

Subcategories	Categories	Themes	Dimension
Time consuming care	Considerable personal investment	Does the benefit of maintenance care outweigh the cost?	When maintenance care is of low value
Cost demanding			
Questionable benefit of care	Is it worth it?		
A sense of low value			
Only one aspect of a wider need			
Perceived as unavailable	Limited accessibility	Is maintenance care accessible?	
Logistical challenges			
Inherent cultural and social beliefs	Perceived as separate from mainstream care		
Not part of the system			
Lack of knowledge regarding MC			
Intimacy and personal space	A feeling of inadequate patient-doctor relationship	Is maintenance care being delivered in a way which is congruent with a patient-centered perspective?	
Communication, trust and report			
Sensation of retention			
Undesired reaction	Unpleasant feelings and experiences associated with care		
Fear of treatment			

### Does the benefit of maintenance care outweigh the cost?

This theme was constructed from participants’ experience of barriers associated with a *considerable personal investment*. Interviewees felt that participation in MC demanded excessive commitment in terms of time and costs. The sheer number of potential visits raised concerns about time commitments, while worries about cost were mainly associated with the amount of money spent during each visit and were therefore closely related to the cost demand per time unit. Participant 16 provides an example of this:

/…/ to set aside time, to simply get away (from work/everyday life) is a barrier. Add to that the cost. It is fairly expensive for a short treatment session. You might be there for roughly ten minutes for a fairly large amount of money.”

(Participant 16, DYS).

Participants mentioned the need to feel that something was wrong with their musculoskeletal health in order to motivate them to engage in MC. Additionally, the participants indicated that they did not view MC as the one and only solution to their musculoskeletal health issues. Instead, they stressed that MC might potentially constitute one aspect of their health care routine, but that its utility &/or scope of practice was somewhat limited.

/…/ in my opinion, some visits felt unnecessary as I didn’t feel I had any problems”

(Participant 17, AC).

”There was maybe major emphasis on the spine and less focus on muscles and exercise, which didn’t come through as clearly. With this in mind, even if I felt like maintenance care helped me, perhaps a balance or combination of these would have been good.”

(Participant 6, ID).

### Is maintenance care accessible?

This theme arose from participants’ experience of *limited accessibility*. The perception of unavailability included multiple aspects such as no or few chiropractors within close proximity, lack of available treatment times, and difficulty finding a good clinician. Additionally, logistical challenges such as difficulty in physically accessing the clinic were also mentioned as barriers to engaging in and maintaining a care plan.

”Well, it can be difficult to find available treatment times if the chiropractor is popular. I perceived this as a challenge, to find treatment times which suited my work schedule.”

(Participant 7, ID).

Furthermore, patients described a lack of support from their employers about having time off work to attend treatment. Participants often took up these logistical challenges such as lack of time or perceived need, for example:

”There is an issue with time, taking time off work and potentially losing income in order to receive treatment which the body might not desperately need.”

(Participant 17, AC).

The second component of this theme pertained to MC being perceived as separate from mainstream health care. Patients expressed distinct inherent cultural and social beliefs which related to antiquated ideas about the chiropractic profession and treatment methods. Such beliefs appear to be present among other health care providers as well as in society at large:

“A physician that I consulted regarding my back problem, after I had just been to see the chiropractor, asked me in what way I thought my back problems had improved. And I told him that I’d seen a chiropractor, and it was like…I might as well have said that I’d taken poison or something to that physician. He was not at all impressed and started to discuss issues, why it is dangerous and so on...”

(Participant 13, DYS).

Pursuing this further, patients pointed out that MC was not integrated into mainstream health care and described this as a *system fault.* This was linked to multiple factors such as minimal financial support from the government, the chiropractic profession as an outsider, and a dissonance between private and public chiropractic care.

”I think that if maintenance care was integrated into the general health care system, perhaps it would work better. At present, maintenance care is on its own, outside the system.”

(Participant 11, DYS).

Finally, participants felt that a lack of understanding of the concept of MC was a potential barrier to engaging in and maintaining such a care plan. This lack of understanding was about both the chiropractic profession and MC in particular. Patients pointed out that people sometimes do not even know that MC is an option.

”Many people might not even know that maintenance care is an option. Since it’s not widely available here, few people talk about it and such things, and I believe it is due to a lack of accessibility and knowledge which needs to be addressed. At least in this part of the country, most people don’t know what a chiropractor has to offer. I think this is a barrier, people don’t know what maintenance care is and therefore they think they don’t need it.”

(Participant 13, DYS).

### Is maintenance care being delivered congruently with a patient-centered perspective?

The theme relating to whether MC is congruent with a patient-centered perspective derives from statements about inadequate *patient-doctor relationships* and *unpleasant feelings and experiences associated with care,* such as fear of adverse treatment reactions. Participants raised issues about lack of communication, trust and professional rapport between clinician and patient. When asked about being given care-related information, participant 23 answered:

”Almost non-existent. This was probably one of the main reasons I decided to not continue with maintenance care.”

(Participant 23, AC).

”Treatment can be a bit uncomfortable as well. The loud noises and cracks while treating the neck can be very unpleasant.”

(Participant 5, ID).

Further issues identified as potential barriers were related to personal space and intimacy, for example discomfort with physical contact, getting undressed in front of the clinician and the gender of the clinician. Concerns were raised about the suspicion that the chiropractor was trying to keep them under their care against their own preference.

”From time to time he was a bit too personal. Also, he could be a bit too intimate and close at times in a way that I felt uncomfortable with.”

(Participant 6, ID).

”If you have visited the chiropractor maybe eight or nine times, and you feel that “no, things are good”. At that point they sort of wanted me to keep coming, even though I felt done and didn’t want to. I felt like I could manage on my own. This was slightly negative.”

(Participant 19, AC).

### Contrasting

Data analysis demonstrated if the different groups as defined by the MPI instrument (adaptive copers, interpersonally distressed, and dysfunctional), mentioned the different subcategories relating to facilitating factors (Table 4) and barriers (Table 5) for engaging and maintaining a MC plan. As such, a contrasting analysis established differences and similarities between the different groups.

**Table 4:** Subcategories relating to facilitating factors for engaging in and maintaining a maintenance care plan, according to which ones were mentioned by participants in each group

Subcategories: facilitating factors	AC group	ID group	DYS group
It made my pain go away	✓	✓	✓
Enables me to stay well over time	✓	✓	✓
My physical abilities have improved	✓	✓	✓
Stimulated healthier behaviors	✓	✓	✓
Allows me to enjoy life	✓	✓	✓
Helps me with my emotions, thoughts & boosts my self-confidence	✓	×	✓
Avoiding sick-leave	✓	×	✓
Being more productive at work	✓	×	✓
Readily available care	✓	×	✓
Time efficient & effective treatment	✓	✓	✓
Small invested effort & no hassle	×	×	✓
Societal or employer reimbursement	×	×	✓
Regular visits offered continuity and motivation	✓	✓	✓
It created a feeling of reassurance	✓	✓	✓
Complements other health actions	✓	×	×
A sense of professional, caring and personal relationship	✓	✓	✓
Provided me with information, guidance & education	✓	✓	✓

✓, mentioned; × , not mentioned; AC, Adaptive Coper profile; ID, Interpersonally Distressed profile; DYS, Dysfunctional profile

**Table 5:** Subcategories relating to barriers to engaging in and maintaining a maintenance care plan, according to which ones were mentioned by participants in each group

Subcategories: barriers	AC group	ID group	DYS group
Time consuming care	×	✓	✓
Cost demanding	✓	✓	✓
Questionable benefit of care	✓	✓	✓
A sense of low value	✓	✓	✓
Only one aspect of a wider need	✓	✓	×
Perceived as unavailable	✓	✓	✓
Logistical challenges	✓	✓	✓
Inherent cultural and social beliefs	✓	×	✓
Not part of the system	✓	✓	×
Lack of knowledge regarding MC	✓	✓	✓
Intimacy and personal space	✓	✓	✓
Communication, trust and report	✓	✓	✓
Sense of retention	✓	×	×
Undesired treatment reaction	×	✓	✓
Fear of treatment	✓	✓	✓

✓, mentioned; × , not mentioned; AC, Adaptive Coper profile; ID, Interpersonally Distressed profile; DYS, Dysfunctional profile

With regard to facilitating factors (Table 4), all three groups mentioned both subcategories relating to the theme *Care that is patient-centered!* Additionally, the dysfunctional group mentioned all subcategories except *Complements other health actions*. In contrast, the ID group mentioned the fewest facilitating factors. The adaptive copers and interpersonally distressed groups did not mention two subcategories related to the theme *Care that is structured, accessible & appreciated!* (*Small invested effort & no hassle* and *Societal or employer reimbursement*).

When it comes to perceived barriers (Table 5), all three groups mentioned most of the barriers, for example *Cost demanding*, *A sense of low value*, *Perceived as unavailable* or *Fear of treatment*. However, the dysfunctional group did not mention the subcategory *Only one aspect of a wider need,* while the adaptive copers group did not mention *Time consuming care* but did mention *Sense of retention*. Finally, during data collection it became obvious that all groups had limited understanding of the concept of MC or even the chiropractic profession.

## Discussion

To the best of our knowledge this is the first study that explores the experiences of patients who have received MC. The aim was to investigate factors which either facilitate or obstruct the procedure. Two main dimensions were inductively generated: when MC is of high or of low value to patients.

Patients who found MC to be of high value said that the procedure improved their quality of life. They felt that the care was structured, accessible, appreciated and delivered with a patient-centered perspective. On the other hand, patients who said MC was of low value questioned whether the benefit outweighed the cost, perceived it as inaccessible and not delivered congruently with a patient-centered perspective.

When contrasting the data with the MPI subgroups as a screen, all subgroups stated that a good relationship with the chiropractor and an appropriate doctor-patient relationship were important. The dysfunctional subgroup mentioned all the facilitating factors, which is in line with earlier research, because they seem to have the best response to MC [4, 6]. Interestingly the interpersonally distressed group mentioned the fewest facilitating factors and stood out by having the shortest interviews and least rich data. The adaptive copers, thought to have the best coping strategies, did not mention “*Societal or employer reimbursement*” or*”Small invested effort & no hassle”* as important facilitating factors, suggesting that this group depended on independent strategies, and that they are more used to taking matters into their own hands rather than relying on others. It is possible that the adaptive copers perceive MC and preventive strategies as a personal responsibility rather than something which should be provided by society.

When it comes to barriers, the three subgroups were more similar compared to facilitators. All subgroups identified cost, logistical challenges and lack of effectiveness as barriers to engaging in and following a MC treatment plan. An additional interesting observation was that most of the participants had a very poor understanding of what MC was, even though they had all received the intervention during the RCT. This was possibly due to poor communication by the treating clinicians. The adaptive copers subgroup stands out in that they did not report”*Time consuming care*”. They did, on the other hand, mention*” Sense of retention”* as a barrier, perhaps a reflection of the fact that they had the poorest outcomes from MC and the least need for a structured and long-term care plan. The dysfunctional subgroup did not mention”*Only one aspect of a wider need”* as a barrier. This is possibly a reflection of the fact that the procedure had the highest effectiveness and greatest utility in this particular subgroup.

In the previous study by Bringlsi et al. [11], based on quantitative data, it was found that patients mainly considered the purpose of MC to be secondary or tertiary prevention. This is in line with the findings from this study, where most of the subthemes relates to prevention or management of their pain condition. The randomized design in the original RCT [4, 6–8] and the qualitative data from this study complement the findings from the previous study [11] by highlighting when MC is of high value and of low value. As we have captured individuals who were randomized to MC in the original trial [4, 6–8] and did not actively choose or engage in MC like the subjects in the Bringsli study [11], we have been able to capture a wider understanding of what drives patient satisfaction and the possible variables associated with choosing or not choosing MC.

**Fig. 1:**
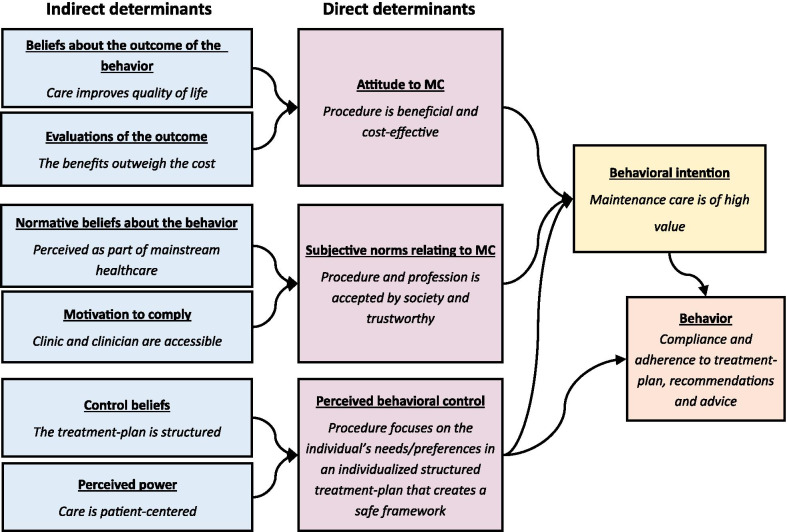
Conceptual behavioral maintenance care model based on the theory of planned behavior

Previous research has indicated that chiropractors perceive MC as beneficial and useful [9, 10, 14–16, 18, 52, 53]. What patients think and the intentions that drive their decision to participate in a course of MC has not previously been studied. Using a theory-based approach it was possible to explore the determinants of human behavior systematically. By using TPB as a conceptual framework to understand patients’ social behavior in relation to MC, the reported facilitators and barriers described in this project may explain patients’ behavior. One central determinant of behavior according to the TPB is the individual’s intention to perform it. In Fig. 1 we have outlined a possible conceptual pathway and behavioral model in which the TPB can be used as a lens through which to understand what governs compliance and adherence to treatment plans, recommendations and patient advice. The TPB fits well with the qualitative data from this study and may be a suitable theoretical framework to use when implementing the procedure in clinical practice. Structuring the information in this way may help the clinician to systematically construct procedures and communication strategies in a way that provides care that is perceived as of high value by the patient.

The knowledge gained from this project may be useful for helping clinicians understand the value of MC. By extension, this may help future research to capture constructs and outcomes closely related to what patients themselves regard as important. Understanding the patient perspective will aid the implementation of the procedure in clinical practice.

A main strength of the study is the purposeful sampling strategy which took sex, age and psychological profile into consideration in order to produce a rich and representative sample. The open and calm interview situation resulted in an expansive data set for the analysis. The richness of the data, together with frequent debriefing sessions and investigator triangulation, enhanced the credibility of the findings [30, 54]. No software packages were used to support the analysis, as we deliberately sought to explore the underlying meaning in the data and consequently performed analysis mainly through iterative peer discussions. Efforts were made to provide rich descriptions of the context and relate the findings to the theoretical framework of TPB to enable transferability of the results to similar settings in which the reader would form part of the validating process [55, 56]. The interpretation of our findings was subject to the same limitations as all small-scale qualitative work. As qualitative research deals with detailed, in-depth analyses and resides within the constructivist paradigm, as opposed to large-scale population-based studies residing within the post-positivistic paradigm, it is neither possible nor desirable to generalize the findings. However, the explicit description of the contextual setting, the participants, and the analytical procedure, together with the links drawn between the findings, the theory and the available scientific literature, may make it possible for the reader to transfer and appraise the applicability of our findings. The chief weakness of the study was the long interval between the study period and the interviews, possibly resulting in some distortion of the data where specific details of participants’ experiences were concerned. As MPI, RMDQ and TDP was not re-assessed 2020 it is likely that both levels of pain, activity limitation and psychological profile were different when the interviews were conducted. However, the purpose of the study was to explore the patients’ pain experiences and perspectives specifically around the study period as this was when they were first introduced to MC and the background data was used to frame this context, not the current state.

Future studies could explore the findings of our constructed themes to find commonalities with other patient cohorts and expand on the derived sub-themes and themes when investigating other MC programs. Such populations may include patients older than 65 and younger than 18 as well as individuals with different socioeconomic backgrounds. The population studied here originally sought care for LBP and future studies should also focus on patients with other pain conditions as well as populations in athletic/sports-oriented settings where physical performance and ability may be of higher value.

Future research should also focus on developing a clinical decision-making tool to select the most appropriate patients for MC [57]. Comparing exercise and MC in an implementation trial would make it possible to test the conceptual TPB model developed in this project and compare the constructs relating to fidelity and compliance of the procedure. In this way we could estimate the correlations between the indirect and direct determinants with specific behaviors relating to successful long-term management of pain.

## Conclusion

The current study addressed how patients experienced MC. It focused on barriers to and facilitators of engaging in and maintaining this kind of care plan and it compared the experience of contrasting psychological subgroups, namely adaptive copers, interpersonally distressed, and dysfunctional. The findings reveal clear positive and negative experiences of MC as expressed by patients across the three psychological subgroups. The findings can contribute to a deeper understanding of MC and can help clinicians and researchers to identify care that patients perceive to have high value.

## Supplementary Information

**Additional file 1**. Interview guide for the project.

## Data Availability

The data generated and analyzed during the current study are not publicly available due to the risk of breaching study participants' data anonymity.

## Abbreviations

LBP: Low back pain
MC: Maintenance care
TPB: Theory of planned behavior
PBRN: Practice-based research network
M: Male
F: Female
MPI: West Haven-Yale multidimensional pain inventory
ID: Interpersonally distressed profile
DYS: Dysfunctional profile
AC: Adaptive coper profile
RMDQ: Roland Morris disability questionnaire
TDP: Total number of days with activity limiting low back pain during RCT study period (52 weeks).
RCT: Randomized clinical trial
