# Supplementary material for: The nordic maintenance care program: patient experience of maintenance care—a qualitative study
Source: Chiropr Man Therap. 2021 Aug 2;29:28. doi: 10.1186/s12998-021-00388-z (PMC8327445; doi:10.1186/s12998-021-00388-z)
Supplement: Supplementary file 1 — Additional file 1. Interview guide for the project. [file 12998_2021_388_MOESM1_ESM.docx]

# Interview guide for the project ”Development of a clinical stratification instrument to improve effectiveness and cost-effectiveness of Chiropractic Maintenance Care”

## Introduction of the project and the interviewer

- **Purpose**: The overarching purpose of the research projects is to develop a clinical instrument designed to identify patients where preventive manual care and rehabilitation (MC) is most effective and cost-effective.
- In this study we set out to investigate patients experiences of MC after partaking in the clinical trial that was conducted by KI during 2012-2016.
- Questions about the project?
- Informed consent (oral and written)

## Introductory questions

## Create confidence and stimulate a relaxed interview environment. Collect demographical data and contextualize.

- Brief description of who they are?
  - Gender
  - Age
  - Marital status
  - Education
  - Work
    - Profession
    - Type of work, seated, standing or moving?
    - On sick-leave?
  - Leisure activities
  - Physical activity
    - How many days per week do they exercise?
    - General physical activity?

## Background questions

### Explore and identify beliefs about underlying cause of their low back problem and knowledge about Maintenance Care.

- Ask about their current or previous low back problem?
- How did they get in contact with their chiropractor?
- What is their general experience of chiropractic care?
- Have they heard about the concept chiropractic maintenance care or as we will call it in this project preventive chiropractic care?
  - If the participant haven´t heard of MC, explain so the participant understands in order for the interview to be able to proceed.

## Questions about attitudes and preferences

- Overall perception of/attitude to preventive chiropractic care?
- Description of preventive chiropractic care with a few words?
- After the study at KI had finished did they continue with preventive chiropractic care, if so how long?
- If they stopped with preventive chiropractic care, why?
- If they did continue with preventive chiropractic care, why?
- Would they recommend preventive chiropractic care to someone else?

## Facilitating factors

## Focus on enabling circumstances and positive experiences.

- How has preventive chiropractic care helped/been beneficial?
- Can preventive chiropractic care prevalent ill health/pain/dysfunction?
- Advantages with preventive chiropractic care specifically?
- Other advantages, more generally with preventive chiropractic care?

## Barriers

## Focus on hindering circumstances and negative experiences.

- Disadvantages and negative experiences with preventive chiropractic care?
- Single most important barrier to partaking in preventive chiropractic care?
- Any other disadvantages or negative aspects of preventive chiropractic care?

## Other comments

## Open questions where the participant is free to talk about their experiences in whatever way they like.

- Anything else to add?

## Thank the participant for their contribution in the study and end the interview.
